# Brassinosteroid-lipid membrane interaction under low and high temperature stress in model systems

**DOI:** 10.1186/s12870-022-03619-4

**Published:** 2022-05-19

**Authors:** Elżbieta Rudolphi-Szydło, Barbara Dyba, Anna Janeczko, Dariusz Latowski, Iwona Sadura, Maria Filek

**Affiliations:** 1 Institute of Biology, Pedagogical University, Podchorążych 2, 30-084 Kraków, Poland; 2 Polish Academy of Sciences, The Franciszek Górski Institute of Plant Physiology, 30-239 Kraków, Poland; 3 Department of Plant Physiology and Biochemistry, Faculty of Biochemistry, Biophysics and Biotechnology, Jagiellonian University, Gronostajowa 7, 30-387 Kraków, Poland

**Keywords:** Brassinosteroids, Homocastasterone, Castasterone, Cell membrane, Langmuir technic, plant, EPR method, electrokinetic potential

## Abstract

**Background:**

In earlier studies [1], we indicated that applying brassinosteroids (BRs) to lipids that had been isolated from plants altered the physicochemical properties of the monolayers. A continuation of these dependencies using the defined model lipid systems is presented in this paper. The influence of homocastasterone (HCS) and castasterone (CS) (BRs for which the increase in concentration were characteristic of plants grown at low temperatures) on the membrane properties of their polar and the hydrophobic parts were studied.

**Results:**

Changes in the electrokinetic potential indicate that both BRs decreased the negative charge of the surface, which is an important factor in modifying the contacts with the polar substances. This property of BRs has not yet been described. The studies of the interactions that occur in the hydrophobic part of the membrane were investigated using the EPR methods and Langmuir techniques. The physicochemical parameters of the lipid structure were determined, and the excess of Gibbs free energy was calculated.

**Conclusion:**

We conclude that examined BRs modify both the hydrophilic and hydrophobic properties of the membranes, but to a greater extent HCS. The consequence of these changes may be the attempt to maintain the stability of the membranes in stressful temperature conditions and / or to the possibility of adsorption of other substances on membranes surfaces. The change of plant metabolism towards increasing the amount of BR, mainly HCS (under cooling) may by an important factor for maintaining optimal structural properties of membranes and their functionality despite temperature changes.

## Background

The impact of temperature stress (both low and high temperature) on a quantitative and qualitative drop in yields as well as disturbances of the physiological processes of plants are well‐documented [2, 3]. However, a detailed description of the biochemical/biophysical stages that guide this process still needs to be provided. Understanding the mechanism of the responses of plants to a change in temperature is critical in order to anticipate the influences of climate change on their production and to develop resistant varieties.

When plant cells are exposed to even slight changes in temperature, specific rearrangements of the lipid composition of the membrane prevents a membrane rupture during the process of hypo-/hyperosmosis [4, 5]. It was indicated that during the acclimation of plants to temperature stress, a crucial process is the modulation of membrane fluidity [6–8] via changes in the unsaturation of the fatty acids in the hydrophobic part of the membrane [6, 7, 9, 10]. When the changes in the hydrophobic part of lipids in plants that have been exposed to a low temperature were profiled, it was revealed that there is usually a significant increase in the content of the lipids with unsaturated fatty acids, especially 18:2 and 18:3 [1, 11–14], whereas a high temperature caused an increase in the amount of the less unsaturated 16:0, 18:0 and 18:1 fatty acids [1, 15, 16]. These dependencies were found independent of the kind of the polar part of the lipid that was esterified with the fatty acids.

It is generally accepted that the overproduction of reactive oxygen species (ROS), which are generated of the stress factor, is an important factor in the stress mechanism, leading to disruption of the biomolecules, including the membrane lipids [17–19]. Stress‐induced lipid peroxidation and changes in the membrane lipid structure are responsible for membrane damage, electrolyte leakage and cell death [20]. Lipid peroxidation can disrupt the ordering of the membrane system. Model studies performed for phospholipids, a group of lipids that are present in all types of membranes, showed that those that contained oxidised fatty acids with more polar head groups might migrate towards the water phase, which leads to an increase in the mean lipid molecular area, and a decrease in the lipid bilayer thickness [21–23]. Detailed detections indicated that the oxidation of phospholipids caused the appearance of pores and a reorganisation of the lipids in some parts of a membrane [17, 24]. Megli et al. [25] point to that the presence of an oxidatively shortened molecule of phosphatidylcholine in mixtures with cholesterol promoted the occurrence of the cholesterol-rich domains such as those that were observed in plasma membrane rafts. Such a specific organisation of lipids and/or lipid/other amphiphilic molecules to domains in stress conditions could also be one of the steps in cell signaling towards the adaptation to stressful conditions [26].

Based on the physicochemical characteristics of the possibility of creating microdomains in the membranes, the presence of two lipid phases was suggested and defined as liquid-ordered (L_o_) and liquid-disordered (L_d_) phases [27, 28]. Klotzsch and Schütz [29] postulated a relationship between the redistribution such specific composed lipid structures in a membrane and a modification of its fluidity. Detailed calculations of lipid-lipid interactions indicated lower diffusion constants in the L_o_ than in the L_d_ phase, which suggests a greater stabilisation of the bonds between the molecules in the L_o_ region. This relationship may also be applied to domains that contain other amphiphilic substances in addition to lipid, which may potentially exist in membranes.

For mixtures of lipids with cholesterol, which were selected in order to mimic the structure membranes in mammalian cells, a dependence between the increasing concentration of cholesterol and the possibility of the formation of L_o_ domains in both the lipid bilayers and monolayers was revealed [29, 30]. These studies provide the basis for the conclusion that the incorporation of other sterol-like substances into a membrane might also affect the formation of the domains in the membrane, thus leading to a modification of its fluidity.

In plant cells, the stabilisation of the membrane structure is conditioned by the presence of steroidal molecules. In studies of the cellular metabolisms, it has been shown that quantitative and qualitative changes in the composition of steroids such as brassinosteroids (BR) accompanied the cultivation of plants at both higher and lower temperatures relative to 20 °C [3, 31, 32]. It has been suggested that the group of these hydrophilic/hydrophobic substances is an important factor that is responsible for increasing the tolerance of cells to stress conditions [33–36]. Although more than 80 brassinosteroids are known [37], but the species, cultivars and even organs (leaves, seeds) differ in their profile and content of BR [32, 38, 39]. Generally, in the leaves of cereals, the dominant BR are homocastasterone and castasterone. Cultivars of wheat and barley that had a higher content of these brassinosteroids (especially homocastasterone) were more tolerant to frost after cold hardening [31, 32]. On the other hand, a lower accumulation of homocastasterone after acclimation at 27 ℃ in barley plants correlated with higher tolerance to extreme temperatures of even more than 40 ℃ [31]. On a cellular level, BR are accumulated in the chloroplasts as has been shown for wheat [40] and barley [41]. However, also in these cases, differences between the species, temperature of plant growth and stress conditions markedly changed the BR content/profile in these organelles. The group of these BRs differed in their chemical structure (i.e., the number of polar OH and/or O groups, or a longer or shorter hydrophobic part) with the overall content of carbon rings (with 27–29 C) [42–44]. The amphiphilic character of BR suggested the possibility of the incorporation of these substances into the lipid part of membranes, which was also confirmed in model research [13]. Thus, it can be assumed that the genotype-dependent fatty acid composition of membranes may be an important factor in the possibility of locating the selected BRs (with an appropriate chemical structure). In our previous studies, it was indicated both changes in BRs synthesis (especially castasterone (CS) and homocastasterone (HCS)) [31] and the remodelling of the membrane structures [1] in barley brassinosteroid mutants that had been subjected to low and high temperatures. The chemical structure of the HCS molecule relative to CS only differs in the hydrophobic part, which is extended by the CH_3_ group.

In our previous experiments in which the interaction of lipid membranes with the isomers of another amphiphilic molecule (zearalenone) was studied, it was shown that even small differences in its chemical structure can modify the spatial positioning of molecules, thus leading to their more or less planar orientation in the hydrophobic part of the lipid layer [45]. This prompted us to search for an explanation of whether/to what extent the differences in the structures between CS and HCS determine the possibility of their interaction with the lipids. Török et al. [46] suggested that one of the initial steps in the mechanism of the response to temperature stress are the subtle changes in the membrane structure, and therefore, it can be assumed that the modification of the composition of the membranes by the incorporation of specific BRs into the lipid membranes might constitute an important stage that leads to the adaptation of plant cells to temperature stress.

Therefore, the aim of the undertaken research was to demonstrate the similarities/ differences in the modifications of the structural properties of membranes by two important brassinosteroids – CS and HCS (applied individually and in a mixture), within a range of low and high (relative to 20 °C) temperatures through experiments conducted in model membrane systems. This goal was achieved by: i) determining the electrokinetic potential of liposomes, which allows to conclude on the influence of the studied BRs on the hydrophilic properties of lipids; ii) designating the temperature range in which there was a modification of the fluidity of membranes that was dependent on the presence of BRs using electron paramagnetic resonance (EPR) measurements and iii) calculating the physicochemical parameters of the membrane structure that is conditioned by the occurrence of BRs in the lipids (Langmuir technique). In the study of electrokinetic properties, a model of liposomes that had been constructed from native membranes that had been obtained from barley plants (cultivar Bowman) that were growing at various temperatures were used because both the modification of the fatty acid composition and the content of CS and HCS were previously demonstrated for this genotype [1, 31]. Based on the similarities of the electrokinetic potential changes to the native membranes (polar part of membrane) and the averaged ratio of un-/saturated fatty acids (hydrophobic part), a mixture of phosphatidylcholine (PC 18:3) + phosphatidylglycerol (PG 16:0) (1:1) was selected as the equivalent of natural systems.

## Results

### Changes in the electrokinetic potential of the liposomes

For the liposomes prepared from the phospholipid fraction (PL) of the lipids that had been extracted from the cells of the barley (0) that was grown at 27 °C, the values of electrokinetic potential were only slightly lower (by about 4.2 mV) than those for the barley that had been cultivated at 20 °C (Table 1), whereas those that had been cultured at 5 °C were significantly lower (by about 19.6 mV).

**Table 1:** Zeta potential of the liposomes made of the lipids that had been extracted from the barley cells of cultivar Bowman grown at 20, 5 and 27 ℃; synthetic lipids PC 18:3 + PG 16:0; 1:1 (M:M) and the studied brassinosteroids (BRs): castasterone (CS), homocastasterone (HCS) and mix of CS and HCS

System	Electrokinetic potential [mV]
**Bowman 20 ℃**	
native lipid	-81.6 ± 1.7^a^
Lipid with HCS 4:1	-52.3 ± 2.3^d^
Lipid with CS 4:1	-77.5 ± 2.5^b^
Lipid with (HCS + CS) 4:1	-68.5 ± 2.7^c^
**Bowman 5 ℃**	
native lipid	-101.0 ± 3.0^a^
Lipid with HCS 4:1	-68.3 ± 2.0^c^
Lipid with CS 4:1	-74.1 ± 2.2^b^
Lipid with (HCS + CS) 4:1	-71.2 ± 2.1^b^
**Bowman 27 ℃**	
native lipid	-86.4 ± 2.6^a^
Lipid with HCS 4:	-57.2 ± 1.7^b^
Lipid with CS 4:1	-59.5 ± 1.8^b^
Lipid with (HCS + CS) 4:1	-58.3 ± 1.7^b^
**PC 18:3 + PG 18:1 (1:1)**	
native lipid	-90.0 ± 2.7^a^
Lipid with HCS 4:1	-64.5 ± 1.9^c^
Lipid with CS 4:1	-89.1 ± 2.8^a^
Lipid with (HCS + CS) 4:1	-79.2 ± 2.4^b^
**BRs**	
CS	-14.6 ± 0.4^a^
CS + HCS (1:1)	-5.76 ± 0.2^b^
HCS	-3.11 ± 0.1^c^

Based on the data obtained for liposomes prepared from the PL native membranes, and the results on fatty acid unsaturation [1], the mixture of PC 18:3 + PG 16:0; 1:1 (M:M) is proposed to mimic native system. There were constructed liposomes from the synthetic lipids whose values of potential were similar to those were recorded at 20 °C that contained PC 18:3 + PG 16:0; 1:1 (M:M).

The introduction of BR into the liposomes increased the electrokinetic potential compared to the control independent of the type of BR that was added. However, for cv. Bowman, larger changes were associated with the presence of HCS compared to CS, especially at 5 °C, whereas at 27 °C, the effects of both BRs on the modification of the potential were similar. When both BRs were simultaneously (HCS + CS) present in the liposomes, the electrokinetic potential took values that were closer to those that were obtained for CS. Measurements of the BRs that were dispersed separately or in the mixture in the water medium revealed that the electrokinetic potential values were more negative for CS than HCS, while in the mixture of both, the values of potential were closer to those that were obtained for HCS.

### Determining the influence of brassinosteroids on the molecular dynamics of membranes using EPR spectroscopy

**Fig. 1:**
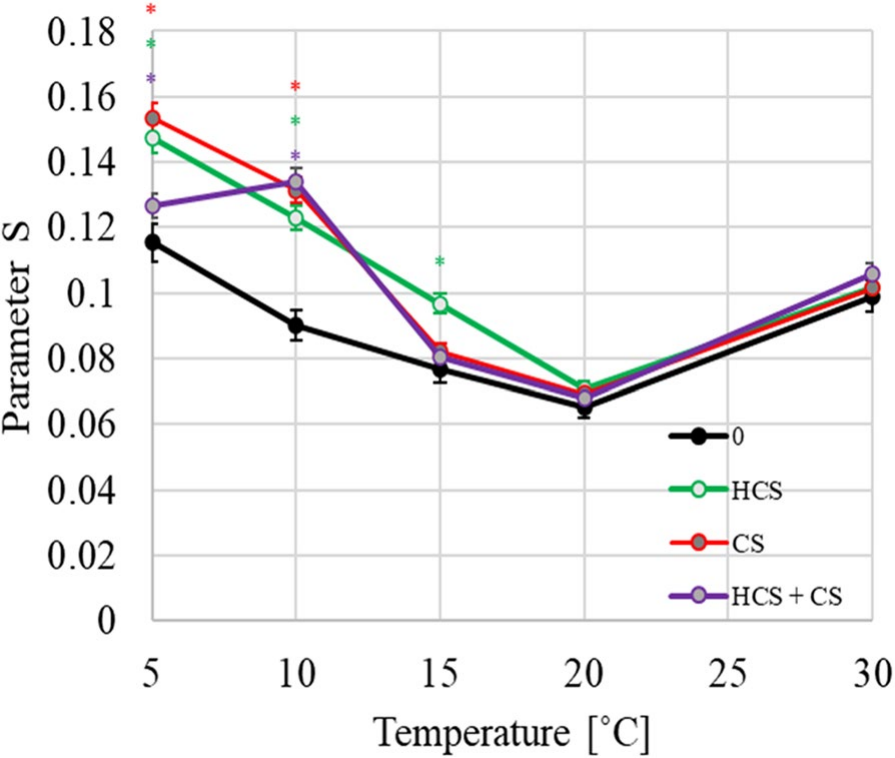
Temperature dependence of the S parameter that was calculated for the PC 18:3 + PG 16:0 liposomes with an embedded 16-SASL marker; measurements were taken at temperatures of 5, 10, 15, 20 and 30 °C. The systems without a BRs (0) were marked by a dark line and the systems with brassinosteroids—by a color lines: homocastaterone (HCS)—green line; castaterone (CS) – red line; homocastasterone + castasterone (HSC + CS) – purple line. Appropriately, mean value (± SD) between the control and the tested system was marked: by green asterisk for HCS, red asterisk for CS, purple asterisk for HCS + CS system (according to Duncan’s test (*P* ≤ .05)

The changes of the order parameter (S) parameter obtained on the basis of Spin Label EPR for the lipids that were composed only of PC18:3 + PG16:0 and for the mixture of lipids with CS and HCS at various temperatures (5, 10, 15, 20 and 30 °C) are presented in Fig. 1.

For each studied system, this parameter generally decreased with an increasing temperature in the range of 5 to 20 °C, whereas it also increased when the temperature was raised to 30 °C. When added individually to the liposomes, both HCS and CS caused an increase in the S value compared to those that were obtained for the lipids without BRs, but the differences were significant only in the range of lower temperatures. For HCS, the range of a noticeable S value increase was at temperatures from 5 to 20 °C, while for CS, it was at temperatures from 5 to 15 °C. When both BRs were simultaneously introduced into the liposomes, the S parameter values at 5 °C were lower than for the systems in which these substances were added individually.

At 10 °C, similar values of the S parameter for the liposomes that contained both BRs were recorded as like that for HCS and CS (independently present), while at 15 °C, the membrane dynamics was similar to that recorded for the pure lipid (Fig. 1a and 1b).

### Calculating the structural parameters of the monolayers that were obtained using the Langmuir technique

#### Monolayers of unsaturated lipids

**Fig. 2:**
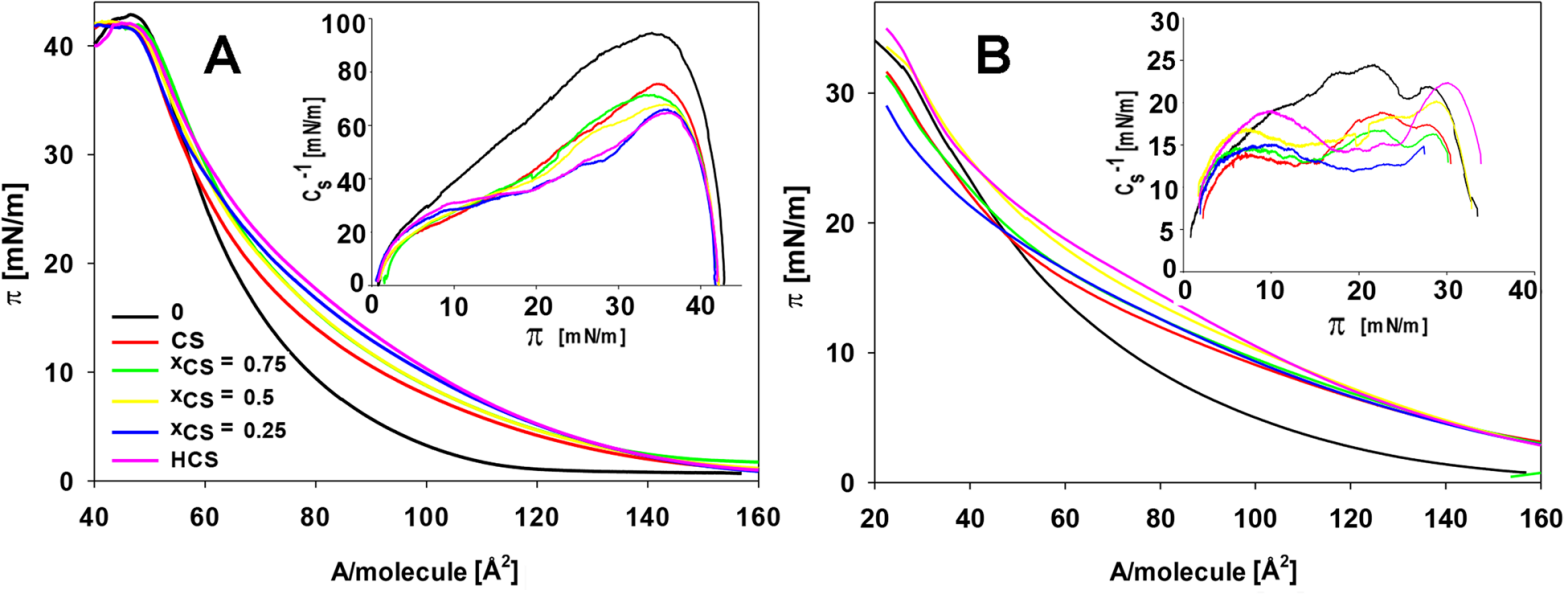
Exemplary Langmuir isotherms (surface pressure **π** vs area per molecule **A**) for the monolayers of the unsaturated phosphocholine – PC 18:3 without BRs (0), with pure castasterone (CS), with a mix of homocastasterone plus castasterone at different molar ratios (**x**_**CS**_ – molar fraction of castasterone in the mixed BRs solution) and with pure homocastasterone (HCS). Measurements taken at 20 ℃ (**A**) and 10 ℃ (**B**). Inserted figure shows the dependence of the compression modulus (C_s_^−1^) vs the surface pressure of the analogous systems

The exemplary isotherms of the surface pressure (π) as a function of the surface area per single lipid molecule in the monolayer (A/molecule) for the PC 18:3 lipid (0) and its’ mixture with BRs are shown in Fig. 2.

Based on these relations, the compressibility modulus was determined and introduced into the internal part of Fig. 2. The presence of BRs in the studied monolayers significantly influenced the shape of the isotherms, especially in the ranges with low π values. These isotherms shown smoother courses (with lower A values higher π values are achieved). The addition of the tested BRs also reduced the value of the C_s_^−1^ compressibility modulus of the studied monolayers. The direction of these changes was similar for all of the BR-containing systems.

Based on the obtained isotherms, the physicochemical parameters that characterized the monolayers were calculated, i.e., A_lim_ – the surface area that was occupied by a single lipid molecule in a maximally packed layer, π_coll_ – the surface pressure at which the monolayer collapsed, which is a parameter that provides information about the stability of the monolayer and C_s_^−1^_max_ – the maximum compressibility modulus, allowing to speculate about the stiffness of the lipid layer. For the monolayers of PC 18:3 (0) and the mixture of this lipid with the tested BRs at various mutual ratios. these parameters are presented in Table 2.

**Table 2:** The effect of two brassinosteroids – castasterone (CS) and homocastasterone (HCS) that were present separately and in mixtures (**x**_**CS**_ – molar fraction of castasterone in the mixed solution) – on the limiting area per molecule (A_lim_ [Å_2_]), collapse pressure (π_coll_ [mN/m]) and maximal compression module (C_s_^−1^_max_ [mN/m]) of the monolayers that had been prepared from phosphocholine (PC 18:3) at 20 °C, 10 °C and 30 °C. Mean values (± SE) marked with the same letters for a measurement were not different according to Duncan’s test (*P* ≤ .05)

Lipid	Applied hormones	A_lim_ [Å^2^]	π_coll_[mN/m]	C_s_^−1^_max_ [mN/m]
	**10 ℃**			
**PC 18:3**	**0**	62.9 ± 0.1^e^	28.2 ± 0.3^c^	24.2 ± 0.5^a^
	**CS**	66.8 ± 0.2^d^	28.1 ± 0,4^c^	18.6 ± 0.8^d^
	**x**_**CS**_** = 0.75**	67.7 ± 0.2^c^	28.6 ± 0,4^b^	16.5 ± 1.3^d^
	**x**_**CS**_** = 0.5**	69.2 ± 0,3^b^	28.8 ± 0,3^b^	19.8 ± 0.5^c^
	_**XCS**_** = 0.25**	72.1 ± 0,4^a^	29.0 ± 0,3^b^	20.0 ± 0.4^b^
	**HCS**	72.4 ± 0,3^a^	29.5 ± 0,4^a^	22.0 ± 0.4^b^
	**20 ℃**			
	**0**	74.3 ± 0.3^f^	42.4 ± 0.3^a^	93.8 ± 0.4^a^
	**CS**	75.8 ± 0.3^e^	40.9 ± 0.4^b^	76.2 ± 0.5^b^
	**x**_**CS**_** = 0.75**	76.8 ± 0.3^d^	40.8 ± 0.5^b^	72.0 ± 0.4^c^
	**x**_**CS**_** = 0.5**	77.3 ± 0.4^c^	40.5 ± 0.6^b^	67.6 ± 0.6^d^
	**x**_**CS**_** = 0.25**	79.3 ± 0.3^b^	40.6 ± 0.5^b^	65.8 ± 0.3^e^
	**HCS**	80.0 ± 0,4^a^	40.6 ± 0.4^b^	64.7 ± 0.5^f^
	**30 ℃**			
	**0**	77.3 ± 0.5^c^	39.9 ± 0.4^a^	77.6 ± 0.4^a^
	**CS**	79.4 ± 0.3^b^	39.6 ± 0.4^b^	70.3 ± 0.5^b^
	**x**_**CS**_** = 0.75**	79.0 ± 0.4^b^	39.9 ± 0.5^a^	70.6 ± 0.6^b^
	**x**_**CS**_** = 0.5**	79.3 ± 0.3^b^	39.9 ± 0.6^a^	66.2 ± 0.6^c^
	**x**_**HCS**_** = 0.25**	80.1 ± 0.2a	40.0 ± 0.5^a^	62.3 ± 0.5^d^
	**HCS**	80.1 ± 0.3^a^	40.5 ± 0.3^a^	62.1 ± 0.4^d^

At 20 ℃, the addition of BRs influenced the increase of the A_lim_ and decreased the C_s_^−1^ values. Although the direction of changes was the same for both BRs, the values of A_lim_ were smaller for CS and for those that were obtained with an increased content of HCS in the lipid monolayers. Analysing the changes of the π_coll_ parameter, it was shown that independent of the kind and proportion, the BRs values of this parameter were similar, but were lower than those for the pure PC 18:3 layers. The direction of changes caused by the presence of hormones for the unsaturated PC 18:3 layers at 10 ℃ and 30 ℃ is the same as at 20 ℃ (being the reference temperature). However, for a temperature of 30 °C, the values that were calculated for both parameters (A_lim_, π_coll_) were more similar to those that were obtained for those at 20 °C, whilst for 10 °C, they were much higher.

#### Monolayers of saturated lipids

**Fig. 3:**
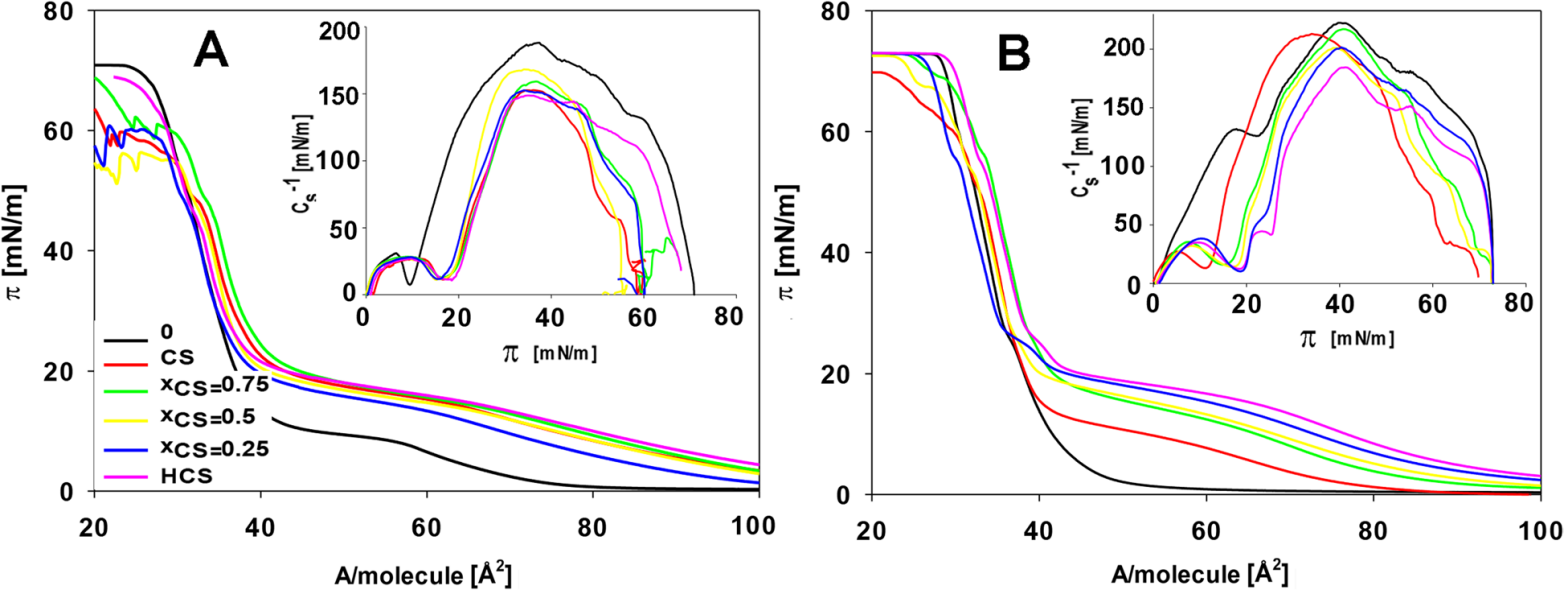
Exemplary Langmuir isotherms (surface pressure **π** vs area per molecule **A**) for the monolayers of the phosphocholine – PC 16:0 without BRs (0), with pure castasterone (CS), with a mix of homocastasterone + castasterone at various molar ratios (present independently and mixed (**x**_**CS**_ – molar fraction of castasterone in the mixed solution) and with pure homocastasterone (HCS). Measurements taken at 20 ℃ (**A**) and 10 ℃ (**B**). Inserted figure shows the dependence of the compression modulus (C_s_^−1^) vs the surface pressure of the described systems

For the PC16:0 lipid monolayers and mixtures of this lipid with the tested BRs, the obtained isotherms are presented in Fig. 3.

The course of the isotherms for PC 16:0 showed the presence of a plateau, thus indicating a phase transition in this region of the monolayer packing. Such a plateau was also visible in the isotherms of the lipids that contained the examined BRs; however, they were shifted, especially relative to the y axis, and therefore, they occurred at the higher values of the surface pressure. The precise calculations of the physicochemical parameters are listed in Table 3.

**Table 3:** The effect of two brassinosteroids – castasterone (CS) and homocastasterone (HCS) – present separately and in a mixture (**x**_**CS**_ – molar fractions of castasterone in the mixed solution) on the limiting area per molecule (A_lim_ [Å_2_]), collapse pressure (π_coll_ [mN/m]), pressure of the plateau (π_plateau_ [mN/m]) and maximal compression module (C_s_^−1^_max_ [mN/m]) the monolayers that had been prepared from phosphocholine (PC 16:0) at 20 °C, 10 °C and 30 °C. Mean values (± SE) marked with the same letters of measurement were not different according to Duncan’s test (*P* ≤ .05)

Lipid	Applied hormones	A_lim_ [Å^2^]	π_coll_[mN/m]	π_plateau_ [mN/m]	C_s_^−1^_max_ [mN/m]
		**10 ℃**			
**PC 16:0**	**0**	40.8 ± 0.2^a^	69.8 ± 0.5^c^	-	221.5 ± 0.4^a^
	**CS**	40.5 ± 0.2^b^	59.7 ± 0.4^f^	19.5 ± 0.4^e^	213.4 ± 0.7^c^
	**x**_**CS**_** = 0.75**	41.0 ± 0.3^a^	65.1 ± 0.6^e^	23.2 ± 0.3^d^	215.4 ± 1.2^b^
	**x**_**CS**_** = 0.5**	39.6 ± 0.3^c^	66.7 ± 0.5^d^	24.4 ± 0.4^c^	201.2 ± 0.6^d^
	**x**_**CS**_** = 0.25**	39.5 ± 0.4^c^	70.7 ± 0.4^b^	25.6 ± 0.5^b^	184.2 ± 0.5^f^
	**HCS**	41.2 ± 0.2^a^	71.4 ± 0.3^a^	26.1 ± 0.4^a^	199.8 ± 0.5^e^
		**20 ℃**			
	**0**	40.1 ± 0.3^c^	62.3 ± 0.3^a^	14.1 ± 0.4^c^	187.8 ± 0.5^a^
	**CS**	41.3 ± 0.3^a^	55.0 ± 0.4^e^	23.7 ± 0.4^a^	152.6 ± 0.4^e^
	**x**_**CS**_** = 0.75**	41.3 ± 0.3^a^	56.0 ± 0.5^d^	23.9 ± 0.3^a^	158.4 ± 0.6^c^
	**x**_**CS**_** = 0.5**	41.5 ± 0.4^a^	55.0 ± 0.6^e^	23.0 ± 0.2^b^	166.0 ± 0.5^b^
	**x**_**CS**_** = 0.25**	40.6 ± 0.3^b^	59.9 ± 0.5^b^	23.2 ± 0.4^b^	148.5 ± 0.4^f^
	**HCS**	40.0 ± 0.4^c^	59.0 ± 0.4^c^	23.2 ± 0.4^b^	151.9 ± 0.6^d^
		**30 ℃**			
	**0**	41.1 ± 0.4^c^	47.1 ± 0.4^b^	34.2 ± 0.3^c^	110.7 ± 0.5^a^
	**CS**	44.6 ± 0.2^b^	48.5 ± 0.6^a^	37.4 ± 0.4^b^	95.9 ± 0.6^b^
	**x**_**CS**_** = 0.75**	44.4 ± 0.4^b^	48.3 ± 0.5^a^	38.3 ± 0.5^a^	110.4 ± 0.4^a^
	**x**_**CS**_** = 0.5**	44.9 ± 0.4^a^	48.1 ± 0.4^a^	38.4 ± 0.6^a^	83.9 ± 0.7^d^
	**x**_**CS**_** = 0.25**	44.9 ± 0.3^a^	48.5 ± 0.3^a^	38.1 ± 0.5^a^	83.6 ± 0.6^d^
	**HCS**	45.0 ± 0.2^a^	48.4 ± 0.5^a^	38.6 ± 0.4^a^	84.6 ± 0.5^c^

The values of the A_lim_ parameters for the PC 16:0 monolayers were similar and were only slightly dependent on the temperature, but were significantly lower than those that were recorded for PC 18:3 (by about 20–30 Å). The presence of BRs in the PC 16:0 monolayers stimulated the changes of the A_lim_, and the C_s_^−1^ values to a lesser extent than those for the PC 18:3 monolayers. At 20 °C, the incorporation of CS caused a slight increase (about 1–1.5 Å) in A_lim_, whereas the HCS did not have any effects compared to the pure lipid. A lower temperature did not influence this parameter for the tested BRs systems relative to the pure lipid, and only for the monolayers that contained HCS + CS (in the range of 0.5 and 0.75) a slight decrease was noted. At 30 °C, the presence of both BRs, which had been added independently or in a mixture, resulted in similar effects (an increase of A_lim_ versus that for PC 16:0). The π_coll_ values for the pure lipid decreased significantly when temperature was increased to 30 °C. In the presence of BRs in monolayers, at temperatures of 10 and 20 °C, there was an additional increase (versus the pure lipid) of these parameter values (especially after the introduction of CS into the lipid),and a progressive raise with an increase of the HCS concentration in the mixture. At the temperature of 30 °C, the presence of BR in the lipid layers reduced π_coll_ (the same regardless of the type of BR).

Analyzing the π values for which the plateau on the isotherm was recorded, it can be concluded that with the lowest π_plateau_ values were obtained for the pure lipid, regardless of the measurement temperature, while the presence of BRs increased these values. For 10 and 30 °C, the highest levels of π_plateau_ were obtained when HCS was introduced into the system, while at 20 °C, similar values were obtained regardless of the type of BRs.

The C_s_^−1^ parameter for PC 16:0 had relatively high values compared to PC 18:3, and decreased with an increased measurement temperature. The presence of BRs in the monolayer generally caused a decrease in the level of C_s_^−1^, especially when HCS was introduced into the monolayer.

#### Monolayers of the mixture lipids with unsaturated and saturated fatty acids

In order to determine the effects of the tested BRs on the monolayers whose hydrophobic properties are the result of the presence both un- and saturated fatty acids, the mixtures of the lipids with the same polar group (phosphatidylcholines) and a different composition of the hydrophobic part (i.e., PC 18:3 and PC 16:0 at a 1:1 molar ratio) were used. The parameters of the surface pressure isotherms are summarised in Table 4.

**Table 4:** The effect of two brassinosteroids – castasterone (CS) and homocastasterone (HCS) – independently and mixed (**x**_**CS**_ – molar fractions of castasterone in the mixed solution) – on the limiting area per molecule (A_lim_ [Å_2_]), collapse pressure (π_coll_ [mN/m]) and maximal compression module (C_s_^−1^_max_ [mN/m]) of the monolayers that had been prepared from the mixture of phosphocholines PC 18:3 + PC 16:0 (1:1) at 20 °C, 10 °C and 30 °C. Mean values (± SD) marked with the same letters of measurement were not different according to Duncan’s test (*P* ≤ .05)

Lipid	Applied hormones	A_lim_ [Å^2^]	π_coll_[mN/m]	C_s_^−1^_max_ [mN/m]
	**10 ℃**			
**PC 18:3**	**0**	66.5 ± 0.3^a^	41.7 ± 0.4^a^	160.6 ± 0.6^a^
** + PC 16:0 (1:1)**	**CS**	65.4 ± 0.1^b^	38.8 ± 0.5^b^	112.9 ± 0.9^b^
	**x**_**CS**_** = 0.25**	64.6 ± 0.3^c^	37.4 ± 0.4^c^	94.7 ± 1.3^c^
	**x**_**CS**_** = 0.5**	62.0 ± 0,2^d^	35.4 ± 0.8^d^	70.0 ± 0.5^d^
	**x**_**CS**_** = 0.75**	60.8 ± 0,2^e^	28.1 ± 0.4^e^	58.3 ± 0.6^e^
	**HCS**	58.6 ± 0,3^f^	27.6 ± 0.6^f^	57.7 ± 0.7^e^
	**20 ℃**			
	**0**	63.4 ± 0.4^f^	43.4 ± 0.4^a^	98.6 ± 0.9^a^
	**CS**	71.4 ± 0.2^e^	43.5 ± 0,4^a^	73.4 ± 0.8^b^
	**x**_**CS**_** = 0.75**	74.5 ± 0.4^d^	43.5 ± 0,3^a^	73.0 ± 0.9^b^
	**x**_**CS**_** = 0.5**	75.1 ± 0.3^c^	41.0 ± 0,2^b^	68.5 ± 1.6^c^
	**x**_**CS**_** = 0.25**	75.4 ± 0.3^b^	41.0 ± 0,3^b^	63.3 ± 1.3^d^
	**HCS**	72.9 ± 0,5^a^	43.7 ± 0,4^a^	61.0 ± 0.9^e^
	**30 ℃**			
	**0**	74.8 ± 0.5^b^	41.7 ± 0.5^a^	89.5 ± 0.7^a^
	**CS**	76.6 ± 0.6^a^	41.4 ± 0.6^a^	72.5 ± 0.6^b^
	**x**_**CS**_** = 0.75**	76.0 ± 0.4^a^	41.4 ± 0.5^a^	70.1 ± 0.5^b^
	**x**_**CS**_** = 0.5**	75.2 ± 0.4^b^	39.6 ± 0.7^b^	65.1 ± 0.8^c^
	**x**_**CS**_** = 0.25**	71.2 ± 0.2^c^	38.3 ± 0.5^c^	64.4 ± 0.6^c^
	**HCS**	70.8 ± 0.5^c^	32,1 ± 0.4^d^	64.8 ± 1.7^c^

The values of A_lim_ for the studied systems were closer to those that were obtained for the PC 18:3 objects. When BRs were introduced into the monolayers at 20 °C, there was an increase in the A_lim_ value and a decrease in π_coll_ and C_s_^−1^. These effects grew with the increase of the HCS content in the monolayer. The same direction of changes in the π_coll_ and C_s_^−1^ values characterised both the low (10 °C) and high (30 °C) temperatures. The values of A_lim_ at 30 °C increased when CS were present in the lipid layer in high amounts.

### Percentage changes in the Langmuir monolayer parameters relative to 20 °C

**Fig. 4:**
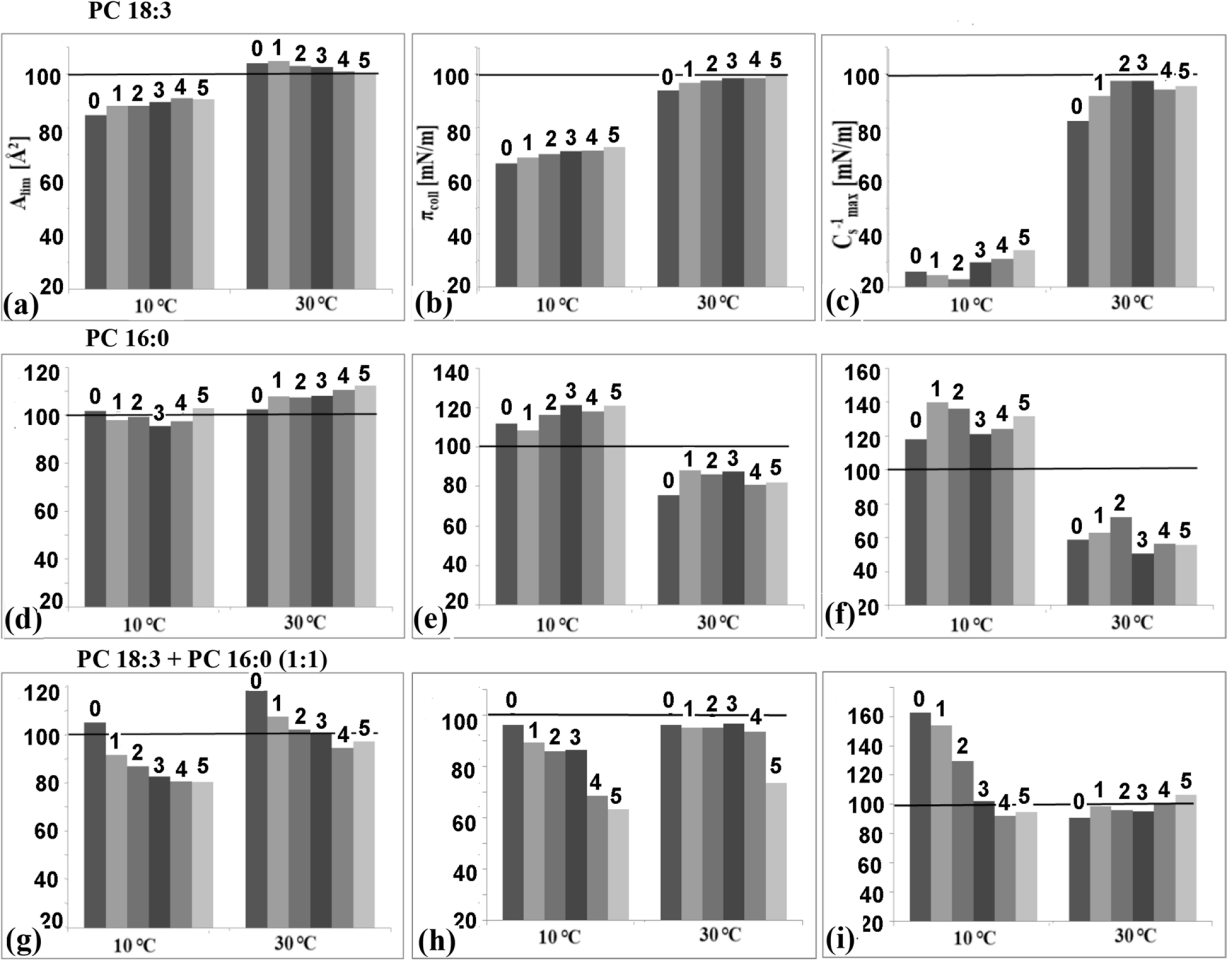
Dependence of the values of the physicochemical parameters of the monolayers that had been prepared from phosphatidylcholines with different saturations of fatty acids on temperature. The results are presented as the percentage values of the data that was obtained at 20 °C (taken as 100% and marked on the graphs as a horizontal line). The graphs use the abbreviations from 0 to 5, which are the symbols of the following monolayer systems: 0 – appropriate PC; 1 – lipid with CS; 2 – lipid with HCS + CS (molar ratio 0.25 HCS:0.75 CS); 3 – lipid with HCS + CS; (molar ratio 0.5 HCS:0.5 CS); 4 – Lipid with HCS + CS (molar ratio 0.75 HCS:0.25 CS) and 5 – lipid with HCS

To better visualise the temperature-dependent changes of the physicochemical properties of the monolayer, all of the isotherm parameters were recalculated as a percentage of the values that were detected at 20 °C (assumed to be 100%) (Fig. 4).

For PC 18:3, a temperature of 10 °C modified the values of these parameters to a greater extent than a temperature of 30 °C. At 10 °C, the values of A_lim_ and π_coll_ decreased (by about 15% and 35%, respectively) and were lower for the pure PC 18:3 (0,) compared to the mixtures with BRs. However, the presence of HCS differentiated the monolayer parameters more than CS versus the pure lipid. The opposite direction of A_lim_ changes for the tested systems was observed at a temperature of 30 °C at which the values for the pure lipid and its’ mixture with CS were higher and decreased with an increase of HCS. At 30 °C, the π_coll_ parameter, similar to the one 10 °C, increased with an increased content of the studied BRs, and was the highest for HCS. When the changes of C_s_^−1^ were analysed, there was a significant decrease in the values at the lower temperature, which was about 75% lower than the values that were obtained at 20 °C. The addition of CS into the lipids additionally reduced the values of this parameter, whereas HCS, increased them. At 30 °C, the presence of the mixture HCS + CS increased the value of this parameter to a greater extent than single the BRs compared to the pure lipid.

For the PC 16:0 systems, the changes of the A_lim_ parameter were smaller. At 10 °C The presence of BR (unlike in the case of the PC 18:3 systems) caused a decrease in this parameter (with the exception of HCS).At 30 °C, there were larger changes in the A_lim_ parameter compared to PC 18:3 and the A_lim_ increased with the addition of HCS into the monolayers. Changes in π_col_ and C_s_^−1^ exhibited different patterns of dependence at low and highs temperature. Compared to 20 °C, at 10 °C, the values of both these parameters increased while at 30 °C, they decreased. In addition, it was observed that the C_s_^−1^ modification (increase compared to PC 16:0) was more influenced by the presence of CS than HCS at both temperatures.

When the percentage changes of isotherm`s parameters for the system PC 18:3 + PC 16:0 were analysed, it was found that at a temperature of 10 °C, the addition of BRs lead to a decrease in the values of all of the parameters that were tested (except for C_s_^−1^, for the case in which CS was added to the lipid). At 30 °C, the values of π_coll_ for all of the objects, and A_lim_ for layers with a large amount of HCS were lower. The C_s_^−1^ values were slightly different from those that were recorded at 20 ℃ (from 1.5 to about 5% for the controls).

### Effects of brassinosteroids on the Gibbs free energy

The isotherms allowed calculating the Gibbs free energy changes of the PC monolayers that were modified by the presence of HCS or CS, according to the formula of [47, 48]:

$$\Delta {G}^{exc}={N}_{A}{\int }_{0}^{\pi 2}\left({A}_{12}-{x}_{1}1{A}_{1}-{x}_{2}{A}_{2}\right)d\pi$$

where A_12_, A_1_, A_2_ are the average molecular surface areas of the components 1 (HCS) and 2 (CS); x_1_ and x_2_ are the molar fractions of the components of the BRs mixture, N_A_ is the Avogadro constant and π is the surface pressure.

**Fig. 5:**
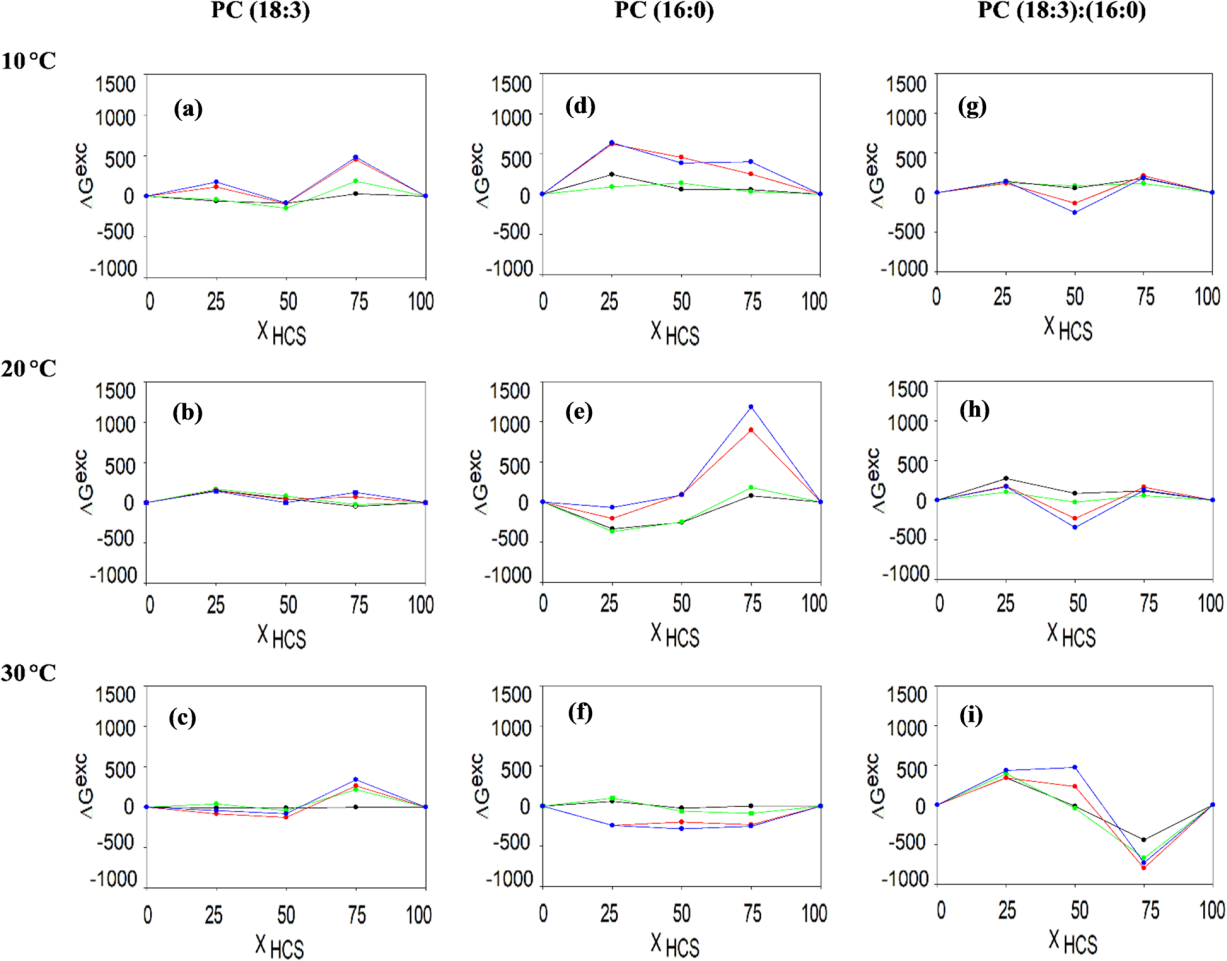
Excess of the Gibbs free energy for the mixtures that were composed of lipid + BRs (at a molar ratio of 4:1; M:M) for the various HCS: CS proportions that were determined for the selected surface pressure (5 mN/m – black line, 10 mN/m – green line, 20 mN/m – red line and for 25 mN/m – blue line). Measurements were taken at three temperatures 10, 20 and 30 °C

The dependencies ΔG^exc^ as a function of the mole fraction of the mixed hormones HCS + CS were calculated from the π-A isotherms for surface pressures equal to 5, 10, 20 and 25 mN/m Fig. 5.

The main focus was on the results that were obtained at an interfacial pressure equal to 25 mN/m at which intermolecular interactions determine the achievement of the monolayer state in which the lipid "arrangement" is closest to that realised in native membranes.

Values ΔG^exc^ above 0 testified to the fact that the interactions between the components in the mixed monolayers are more repulsive than those that occur in a single component monolayer [49, 50]. Thus, a decrease in the value of ΔG^exc^ indicates that the tested systems has become more stable. At 20 ℃ (for all of the tested π), the ΔG^exc^ values for the PC 18:3 monolayers were ± 200, which indicates the stability of all of the tested BR/lipid mixtures. In the case of the PC 16:0 layers, the lowest values of ΔG^exc^ were noted for the system that contained 25% HCS, and the highest were noted for the system that contained 75% HCS. For the mixtures PC 18:3 + PC 16:0, the most stable system was obtained for the mixture CS + HCS 50:50. At 10 °C, for the PC 18:3, an increased ΔG^exc^ was observed for the lipids with 75% HCS, while the lowest values of this parameter were registered when the BRs were present in equal ratios. For the PC 16:0 layers, all of the mixtures had positive values of ΔG^exc^ in contrast to the monolayers of PC 18:3 + PC 16:0, which were characterised by low values of this parameter (especially in the presence of 50% CS). At a temperature of 30 ℃, when BRs were present in the PC 16:0 monolayers, there was a decrease in the ΔG^exc^ value. In the case of PC 18:3, ΔG^exc^ increased in the system that contained 75% HCS. In the monolayers of PC 18:3 + PC 16:0, the lowest values of this parameter were obtained for the presence of 75% HCS.

## Discussion

Changes in the electrokinetic potential of a system offers the opportunity to draw conclusions about the possible occurrence of a charge on the surface of tested objects [49, 51]. The negative values of the potential that were recorded for the phospholipids that had been obtained from the native Bowman barley membranes (0) were consistent with the data that was obtained for other systems that had been constructed from both native and synthetic lipids [1]. A significant decrease in the value of the electrokinetic potential for the phospholipids that had been obtained from plants growing at 5 °C relative to the temperatures of 20 and 27 °C suggest a modification of the polar structure of the membranes towards the incorporation of the negatively charged phospholipids. The remodelling of the membranes in the region of the polar part is one of the effects of stress factors actions’ [52–56]. It was expected that the incorporation of BRs into the lipid liposomes would cause an increase in the electrokinetic potential values because some of lipid molecules were replaced by less negatively charged BRs. In fact, the values of the electrokinetic potential increased after the incorporation of these substances; however, the changes did not represent a simple proportionality between the potentials of the lipids and BRs and were considerably dependent on the pool of lipids that had been extracted from the plants growing at different temperatures. At 5 °C, the PL faction was more enriched with unsaturated fatty acids compared to a temperature of 20 °C [1]. Thus, it is probable that there is a greater possibility of an “easier” organisation of both BRs between the spaces that were created by *cis*-unsaturated fatty acids in these types of the lipid layers (than in those at 20 °C). The consequence of these may be a major influence on the creation of the electrokinetic potential. Interesting results were obtained for the liposomes of the BRs with the lipids that had been obtained from the PL plants growing at 27 °C. For these lipids, the saturation of fatty acids was the highest among the native membrane systems that were tested [1]. However, the decrease in the negative values of the potential, which was induced by the presence of BRs in these lipids, was similar (versus the control) to the one registered at 5 °C regardless of whether they were applied individually or in a mixture. This suggests that the localisation of BRs in the lipid layer is not a simple process that is conditioned only by the hydrophilic/hydrophobic content of membranes, but may be also reliant on a BRs structure-dependent specific orientation between un- and saturated fatty acids. The organization of fatty acids in the hydrophobic part of the membrane may favor sites in the membrane for the absorption of the steroid-like substance and the formation of areas with more brassinosteroids in the membrane structure. The larger changes of the electrokinetic potential that were recorded for the liposomes in which HCS was present compared to those with CS (also in a mixture) suggest that the presence of the BR HCS can modify the electrokinetic properties of the membrane surfaces in the tested systems to a greater extent than CS.

The EPR technique was used to directly measure the BRs-induced changes in the hydrophobic phase of the membranes. The values for the order parameter (S), which were calculated based on the EPR data allowed to speculate about the order of the lipid phase (with S close to 1, which corresponds to the maximal order and S close to 0 for the maximum disorder of the molecules). Generally, the temperature dependence of the course of the changes in the S parameter was similar to the one that was determined by Swamy et al. [27] when they analysed the L_o_ and L_d_ regions in cell lines. The rather low values of the S parameter that were obtained for the tested systems compared to those that were obtained by Swamy et al. [27] for eukaryotic cells indicate a poor organisation of lipids, which is presumably related to a significant amount of unsaturated fatty acids in the tested liposomes (PC 18:3 + PC 16:0; 1:1 (M:M)). The higher S values for the temperatures of 5 and 10 °C compared to 20 °C were as expected because at these temperatures, the ordering of lipids is favoured by a decrease in the kinetic energy of the molecules. On the other hand, the re-increase of the S parameter that was observed at 30 °C could indicate a temperature-dependent specific organisation in the hydrophobic region towards greater molecular order. The presence of BRs in the lipid layer seemed to be “stimulator” of the ordering of the molecules with HCS stabilising this process over a greater temperature range than CS. At low temperatures (5, 10 °C), both BRs seemed to act towards the formation of a more ordered structure of the membranes (than those in the pure lipids) when they were present separately or in a mixture. When these data were analysed in the context of the changes that were observed in the native membranes of the barley plants under colder temperatures (an increase in the unsaturation of fatty acids and the more efficient synthesis of BRs), it can be concluded that the effect of both of these actions could be the blocking of the possible “empty regions”, that were created by the unsaturated fatty acids by the localised hydrophobic BRs parts. This blocking process might be an additional factor that protects membranes in low temperature conditions. The experimentally observed increase in lipid unsaturation is conducive to maintaining the continuity of the membranes (a lower temperature of the fatty acid phase transitions) in cold conditions [6, 7, 9, 10]. The presence of BRs could change the physicochemical properties of the membrane surface, thereby reducing the possibility of penetration by water molecules, the presence of which causes mechanical injuries to the membranes during freezing by increasing the volume of the membrane domains. HCS, which has an additional (relative to CS) hydrophobic substituent could better "fit" into the structure that is formed by fatty acids, which could be the reason for the more efficient synthesis of these BRs relative to CS in barley plants [31].

The study of the changes in the hydrophobic part of lipids under the BRs presences using the Langmuir technique enabled a precise demonstration of those parameters of the structure membranes that differentiate both the temperature- and fatty acids-dependent reactions between HCS and CS in the modification of membrane properties. In agreement with other studies [1], it was shown that the monolayers that are made higher unsaturated lipids (PC 18:3) are characterised by higher surface area values that are occupied by single molecules and less stiffness of the layer compared to the saturated fatty acid monolayers (PC 16:0). This effect is explained as the loosening of the rigid packing of the molecules by weakening the *van der Waals* bonds between the unsaturated acids. Moreover, the lower values of A_lim_ and the higher monolayer stiffness that were obtained at a lower temperature (10 °C) were in line with expectations, which was the result of the temperature-dependent reduction of the mobility of molecules at the hydrophilic/hydrophobic interface. The π-A isotherms of phospholipids usually display two kinds of liquid states of their monolayers, namely a liquid-expanded state (LE) and a liquid-condensed state (LC), which after the maximal compression creates a molecular reorientation to form a compact layer [57, 58]. The linear ("smooth") PC 18:3-BRs isotherms, which had a shape that was similar to the one that was recorded for the pure lipid, indicates that the presence of BR (regardless of the type) did not disturb the course of the isotherm, but only caused subtle changes in the organisation of the molecules in monolayer. Detailed analyses of the surface parameters that were obtained for the HCS and CS that were present in the PC 18:3 monolayers led to conclusion that the molecular space (A_lim_) requirements for both BRs were different, and were higher for HCS. This confirms the earlier results, which indicated a greater influence of this BR (of a more hydrophobic nature) on the properties of the membrane structure. Interesting results were obtained for the monolayers made of PC 16:0 for which the introduction of the tested BRs changed the parameters of the surface structure only slightly. For the measurements that were taken at 10 °C, there was a decrease in the A_lim_ value for the mixed HCS + CS with respect to the pure lipid, which was accompanied by a significant increase in monolayer stiffness (expressed by π_coll_ and C_s_^−1^). High values of these parameters indicate a tightly packed and more ordered layer [59, 60]. A decrease in the A_lim_ in the presence of BRs may indicate a "pushing" of the BRs out of the lipid layer during the condensed monolayer formation similar to the one that was explained for systems in which the fatty acid chain was "shortened" by lipid peroxidation [1, 23, 53]. Another explanation may be the possibility of creating PC 16: 0-BR structures that have an arrangement more similar to the hexagonal packing of molecules, which is similar to that of MGDG structures in lipid bilayers [61, 62]. The presence of such a conformation in the monolayers leads to diminishing A_lim_ compared to the linear packing of molecules [62–64]. Moreover, in contrast to the “smooth” course of the π-A isotherms for PC 18:3, the PC 16:0 isotherms were characterised by the occurrence of a plateau for the systems that contained the tested BRs even at 10 °C, whereas no plateau was recorded for the pure lipid. The presence of a plateau in the course of the pure PC 16:0 isotherm, which indicates the possibility of organising specific lipid domains that determine LC/LE phase transitions, was expected. A plateau shift towards higher π values for the PC 16:0-BRs isotherms indicates that there was a BR-initiated change in the organisation of the lipid domains. The higher plateau values (π_plateau_ values) for the lipid-HCS than for the lipid-CS mixtures, especially at low and high temperatures, indicate that there is a greater contribution of this BR to the formation of more compact lipid structures. The possibility of a different influence of the examined BRs on the properties of the monolayer depending on the unsaturation of lipids was confirmed in the tests that were performed for mixed lipids (PC 18:3 + PC 16:0). The significant influence of HCS on the surface parameters, especially at 10 °C, suggests the possibility of a "remodeling" of specific HCS-lipid structures in the monolayers in order to build a more compact surface of the membranes.

The analysis of the percentage changes of these physicochemical parameters showed that when 20 ℃ was the reference temperature, then the presence of BR in lipids at both the lower and higher temperatures modified the properties of the membranes by shifting them towards those that were obtained at 20 °C. These dependencies indicated that HCS had a much greater impact on the structure of the membranes than CS. This might be the reason for the reactions that were observed in the native membranes (barley) where under the influence of low-temperature stress, the synthesis of HCS was intensified to a greater extent compared to CS [31].

The results that were obtained for the Gibbs energy confirmed the differentiating effect of the examined BRs depending on both the lipid unsaturation and the temperature. For the systems that contained only unsaturated fatty acids (PC 18:3), the variation in the structure of the BRs had a smaller impact on the stability of the monolayers than for the systems that contained only saturated lipids (16:0). This could explain the observed dependencies in the native membranes in which the increase in unsaturation was connected with the synthesis of both of the examined BRs at low temperatures. For the systems with a mixed unsaturation composition, the greater influence on membrane stability in the presence of HCS than CS that was calculated at 30 °C confirmed that for membranes that had a “looser packing” (conditioned by an increase in the kinetic energy of the molecules), the introduction of HCS stabilised the monolayer structure to a greater extent.

## Conclusions

Based on the conducted experiments, we conclude that both BRs (castasterone and homocastasterone) can modify not only the hydrophobic but also the hydrophilic properties of membranes. An increase in the value of the electrokinetic potential of a membrane in the presence of BRs may cause the reorganisation of the ions and polar molecules in its surroundings, thereby changing the molecule-specific tendency to adsorb on the membrane surface (depending on their charge and polarity). This property of BRs is described here for the first time. In the hydrophobic layer, which is rich in unsaturated fatty acids (characteristic for plants grown at low temperature), small differences in the chemical structure between CS and HCS may favour a better "fit" of the latter between the chains of these acids in order to reduce the possibility of the incorporation of other molecules (e.g., water) into the structure of the membranes.

Thus, a change of plant cell metabolism towards increasing the amounts of brassinosteroids, especially HCS (during cooling) and their location in membranes could be important in maintaining the optimal physico-chemical properties of membranes conditioning their functionality despite temperature changes.

## Methods

### Plant material

The barley seeds were obtained from the collection of the University of Silesia (Katowice, Poland). The detailed plant breeding procedure for barley cv. Bowman was previously described [31]. Three-week-old plants (three to four leaf stage) that were grown at 20 °C were separated into two groups. One group was further cultivated for 21 days at 5 °C (8 h photoperiod), while the second group was grown at 27 °C for seven days (16 h photoperiod). The leaves from all of the temperature-treated plants (20 °C, 5 °C and 27 °C) that were at a similar developmental phase (about three to four leaves) were collected and frozen in liquid N_2_ for the lipid analysis.

### Chemicals

Lipids: PC 16:0 1,2-dipalmitoyl-sn-glycero-3-phosphocholine; PC 18:3 1,2-dilinolenoyl-*sn*-glycero-3-phosphocholine and PG 16:0 1,2-dipalmitoyl-sn-glycero-3-phospho-(1'-rac-glycerol) were obtained from (Avanti Polar Lipids, Inc. (USA/Canada)). Brassinosteroids: homocastasterone (HCS) and castasterone (CS) were obtained from Olchemim (Czech Republic laboratory). 16-DOXYL -stearic acid (16-SASL, Sigma-Aldrich, Germany). Solvents: chloroform, ethanol, methanol, isopropanol of chemical purity were purchased from’’POCh’’ (Poland). Freshly deionised water was produced using an HLP 5 apparatus ‘‘Hydrolab’’ (Poland).

### Lipid extraction

Lipids were extracted according to the Blight and Dyer method [65] with the Filek et al. [66] modification using chloroform:isopropanol mixtures (1:1; v:v). The phospholipids were separated from the other polar lipids using column chromatography and the purity of this fraction was controlled by thin layer chromatography [67]. The content of fatty acids was analysed (Trace GC Ultra Thermo Electron Corporation, Milano, Italy) and compared with the data that had been presented by Rudolphi-Szydło et al. [1].

### Liposome preparation

The liposomes were prepared from pure lipids and mixtures with HCS or CS (individually and 1:1 HCS + CS mix) at a ratio of lipid:BRs 4:1 (M:M) according to the procedure that was previously described in [1, 23].

In preliminary studies series of experiments with different doses of hormones was performed. Slight, but statistically significant changes were already noted for the ratio of lipid: hormone mixtures 20:1, however, in further analyzes, in order to more accurately demonstrate the examined physicochemical changes, lipid: hormone mixtures 4:1 were used.

A thin layer of lipid (and lipids with BRs) film was formed on a wall of a round-bottomed glass tube by evaporation (under an Ar atmosphere). The liposomes were created via ultrasonification followed by vortexing in pure, freshly deionised water. The resultant suspension was extruded using a 200-nm-pore polycarbonate membrane.

### Electrokinetic potential determination

A Zetasizer (Malvern Zetasizer Nano ZS apparatus) was used to determine the electrokinetic potential of the liposomes that had been prepared from native and synthetic lipids. This method relies on measurements of the electrophoretic mobility of objects, which is recalculated to the values of electrokinetic potential using the Smoluchowski equation. For each liposome suspension (prepared in three replications), the electrokinetic potential values were determined a minimum of ten times. The data are presented as the mean ± SE.

### Spin label EPR measurements

The molecular dynamics of the membranes was monitored using EPR spectroscopy. 16-doxyl stearic acid (16-SASL), which is a stearic acid derivative was used as the spin label to characterise the dynamics of the interior of the hydrophobic membrane. The 16-SASL molecules were incorporated into the liposome membranes as follows: the methanol solution of 16-SASL was mixed with a lipid solution (PC 16:0 and PC 18:3; 1:1 (M:M) at a final ratio of 0.1 mM:10 mM spin label:lipid [68]. The influence of BRs were analyzed in liposomes prepared from lipids and BRs at a ratio 4:1 (M:M). The EPR spectra of the spin label as a function of the temperature were recorded using an X-band EPR spectrometer (Miniscope, Germany) equipped with a Temperature Controller (Magnettech H02, Germany) in a temperature range of 5 to 30 °C with an interval of 5 °C. The EPR measurements were performed with determined parameters: microwave power between 3.2 and 10.0 mW, a sweep width of 15 mT, a modulation amplitude of 0.1 mT and microwave frequencies of about 9.4 GHz. The values of parameter S were calculated following [68] using the equation [69]:

$$\boldsymbol{S}\mathbf{=0.5407(}\boldsymbol{A}\mathbf{'\parallel-}\boldsymbol{A}\mathbf{'^{\perp})/}\boldsymbol{a}_\mathbf{0}$$

where

$${\boldsymbol a}_{\mathbf0}\boldsymbol=\boldsymbol(\boldsymbol A'\parallel+\mathbf2\boldsymbol A'\boldsymbol)\boldsymbol/\mathbf3$$

### The Langmuir monolayers

The surface pressure isotherms were measured using a Langmuir trough (Minitrough, KSV, Finland) with a Pt-Wilhelmy plate used for surface tension detection (accuracy of ± 0.1mN/m). The Langmuir monolayers were produced by spreading chloroform solutions of the lipids on the surface of a water subphase. The experiments were conducted at three temperatures 10, 20 and 30 ℃. The measurements were repeated three or four times with the high repeatability of the obtained isotherms (0.1—0.3 Å^2^).

Based on the dependence of the surface pressure (π) on the surface per lipid molecule (A), the parameters that describe the structure of the monolayers were determined, i.e., A_lim_ – the area that was occupied by a single molecule in a maximum packed layer and π_coll_ – the value of surface pressure at which the layer collapsed. The compression modulus, which is defined as C_s_^−1^ = -(dπ/dlnA), that represents the mechanical resistance during compression was also calculated. This parameter provides information about layer stiffness.

### Statistical analysis

Data are presented as the mean ± SE. The experiments were repeated at least three times, and each experiment included at least three individual treatments. The data from the various treatments were statistically analysed using the SAS ANOVA procedure, and the means were compared using the Duncan’s test from PC SAS 8.0. Differences of *p* ≤ 0.05 were considered to be significant.

## Data Availability

Not applicable.

## Abbreviations

HCS: Homocastasterone
CS: Castasterone
BRs: Brassinosteroids
EPR: Electron paramagnetic resonance
PC 16:0: 1,2-Dipalmitoyl-sn-glycero-3-phosphocholine;
PC 18:3: 1,2-Dilinolenoyl-*sn*-glycero-3-phosphocholine
PG 16:0: 1,2-Dipalmitoyl-sn-glycero-3-phospho-(1'-rac-glycerol)
16-DOXYL: Stearic acid;16-SASL
A_lim_: The surface area that was occupied by a single lipid molecule in a maximally packed layer
π_coll_: The surface pressure at which the monolayer collapsed
π_plateau_: The surface pressure at which is plateau
C_s_^−^^1^_max_: The maximum compressibility modulus
